# 

**Published:** 1877-10

**Authors:** 


### Contents of October Number.

### ORIGINAL.

ART. I.—Treatment of Diphtheria. By E. N. Chapman, A. M., M. D. 81
ART. II.—Separation of the Ileum and spontaneous Occlusion of the divided extremities. By Prof. Thos. F. Rochester, M. D.,...... 94
ART. III.—Proceedings of the Buffalo Medical Association,........ 96

### CORRESPONDENCE.

Reputation, how obtained,........................................ 98

### MISCELLANEOUS.

Women in the Profession,........................................ 102
Spontaneous Reduction of Inverted Uterus,........................ 105
Infant Mortality and Infants' Food,.............................. 106
Comparative Action of Quinia, Cinchona and Cinchonidia,.......... 107
Medical Reform,.................................................. 108
Dangerous Vinegar,............................................... 108
Prof. Huxley on the Anti-Vivisectionists,........................ 109
Influence of Alcohol,............................................ 109
Hay Fever,....................................................... 110
Death of Prof. Alpheus B. Crosby,................................ 110
Opium Antidotes,................................................. 110
Stem-Pessary,.................................................... 111
A Danger to be Avoided,.......................................... 111

### EDITORIAL.

Education, its influence upon the Eye,........................... 111
Editorial Notes,................................................. 113
BOOKS REVIEWED:
Ziemssen's Cyclopædia, Vol. VIII,................................ 114
Transactions of the American Gynæcological Society............... 115
Atlas of Skin Diseases,.......................................... 117
Diseases of the Skin.—Piffard,................................... 118
Medical Reform,.................................................. 119
Electro-Thermal Bath,............................................ 119
Books and Pamphlets Received,.................................... 120

